# Mental healthcare for young and adolescent LGBTQ+ individuals in the Indian subcontinent

**DOI:** 10.3389/fpsyg.2023.1060543

**Published:** 2023-01-20

**Authors:** Prithvi Sanjeevkumar Gaur, Sreoshy Saha, Ashish Goel, Pavel Ovseiko, Shelley Aggarwal, Vikas Agarwal, Atiq Ul Haq, Debashish Danda, Andrew Hartle, Nimrat Kaur Sandhu, Latika Gupta

**Affiliations:** ^1^Smt. Kashibai Navale Medical College and General Hospital, Pune, India; ^2^Mymensingh Medical College, Mymensingh, Bangladesh; ^3^Department of Medicine, Dr. B. R. Ambedkar State Institute of Medical Sciences, Mohali, India; ^4^Radcliffe Department of Medicine, Oxford, United Kingdom; ^5^Department of Pediatrics, Santa Clara Valley Medical Center, San Jose, CA, United States; ^6^Department of Clinical Immunology and Rheumatology, Sanjay Gandhi Postgraduate Institute of Medical Sciences, Lucknow, India; ^7^Department of Rheumatology, Bangabandhu Sheikh Mujib Medical University, Dhaka, Bangladesh; ^8^Department of Rheumatology and Clinical Immunology, Christian Medical College Hospital, Vellore, India; ^9^Imperial College Healthcare NHS Trust, London, United Kingdom; ^10^Department of Public Health, University of California, Merced, Merced, CA, United States; ^11^Division of Musculoskeletal and Dermatological Sciences, Centre for Musculoskeletal Research, School of Biological Sciences, Faculty of Biology, Medicine and Health, Manchester Academic Health Science Centre, The University of Manchester, Manchester, United Kingdom; ^12^Department of Rheumatology, Royal Wolverhampton Hospitals NHS Trust, Wolverhampton, United Kingdom; ^13^City Hospital, Sandwell and West Birmingham Hospitals NHS Trust, Birmingham, United Kingdom

**Keywords:** COVID-19, LGBTQ (lesbian, gay, bisexual, transgender, queer), adolescent, mental healthcare, sexual and gender minorities

## Abstract

The coronavirus disease (COVID-19) pandemic has led to a significant change in the way healthcare is dispensed. During the pandemic, healthcare inequities were experienced by various sections of society, based on gender, ethnicity, and socioeconomic status. The LGBTQ individuals were also affected by this inequity. There is a lack of information on this topic especially in the developing countries. Hence this issue requires further exploration and understanding. Previous literature briefly explored the mental, physical, and emotional turmoil faced by the LGBTQ community on a regular basis. They feared rejection by family and friends, bullying, physical assault, and religious biases. These issues prevented them from publicly speaking about their sexual orientation thereby making it difficult to collect reliable data. Although they require medical and psychological treatment, they are afraid to ask for help and access healthcare and mental health services. Being mindful of these difficulties, this article explores the various underlying causes of the mental health problems faced by LGBTQ individuals, especially, in the Indian subcontinent. The article also examines the status of healthcare services available to Indian sexual minorities and provides recommendations about possible remedial measures to ensure the well-being of LGBTQ individuals.

## Highlights

- Present condition of LGBTQ + mental healthcare accessibility in the world.
- Present condition of LGBTQ + mental healthcare accessibility in India and neighboring regions.
- Prevalent healthcare disparities faced by the Indian LGBTQ + individuals.
- Reform measures and steps toward providing healthcare to LGBTQ + individuals in the Indian subcontinent.

## Introduction

The word LGBTQ is an acronym for Lesbian Gay Bisexual Transgender and Queer. It is used to describe non-heterosexual and non-cis-gendered individuals. As these terms are often difficult to define, we have utilized the United Nations Human Rights Council (UNHRC) definitions for these terms (Definitions, 2017). These definitions have been provided for reference in Table 1 to provide context and clarity in our discussion. The COVID-19 pandemic has dynamically changed the way healthcare services are being delivered to the public. It led to the worsening of inequitable access to healthcare services based on gender, socioeconomic status, and ethnicity (Lopez et al., 2021). The LGBTQ community has experienced a disproportionate impact of the COVID-19 pandemic with 56% of LGBTQ individuals reporting job losses compared to 44% of the non-LGBTQ individuals. Moreover, 74% of the LGBTQ individuals reported worry and stress related to the pandemic had a negative impact on mental health compared to 49% of the non- LGBTQ individuals (Dawson et al., 2021). There is currently a dearth of data on this topic in developing countries particularly in the Indian subcontinent. Our paper aims to fill this gap in knowledge by examining the mental health issues faced by adolescents who identify as LGBTQ in the Indian subcontinent, their inequitable access to healthcare services, and suggest the possible remedial measures which can lessen the impact of these inequities with a special focus on the COVID-19 pandemic.

**TABLE 1:** Gender identity definitions as per the UNHRC (Definitions, 2017).

Term	Definition
Lesbian/Gay	Attracted to individuals of the same sex and/or gender identity as themselves
Bisexual	Attracted to individuals of the same and opposite sex and/or gender identity as themselves
Transgender	Comprises a wide range of identities including transsexual people, those who identify as a third gender, and others whose appearance and characteristics are perceived as gender-atypical and whose sense of their gender is different from the sex that they were assigned at birth
Queer	Queer is an umbrella term commonly used to define lesbian, gay, bisexual, transgender, and other people, and institutions on the margins of mainstream culture

India legalized gay relationships on September 6, 2018, marking the first step toward recognizing LGBTQ rights in the country (The Times of India, 2018). The first Pride March of India was held in Kolkata in March 1999 (Deccan Herald, 2019). However, there is no reliable systematically collected data on the prevalence of LGBTQ individuals in India as the 2011 Census did not record this data accurately (Mandal et al., 2020). Individuals may be hesitant to come out as LGBTQ due to familial stress, religious bias, fear of rejection, fear of physical assault, cultural stigma, etc., making it difficult to collect reliable data (Centers for Disease Control and Prevention, 2020). The aforementioned factors and others may exacerbate their need to conceal their personal and sexual experiences making them more susceptible to medical and mental health concerns with an insufficient access to healthcare services.

**FIGURE 1:**
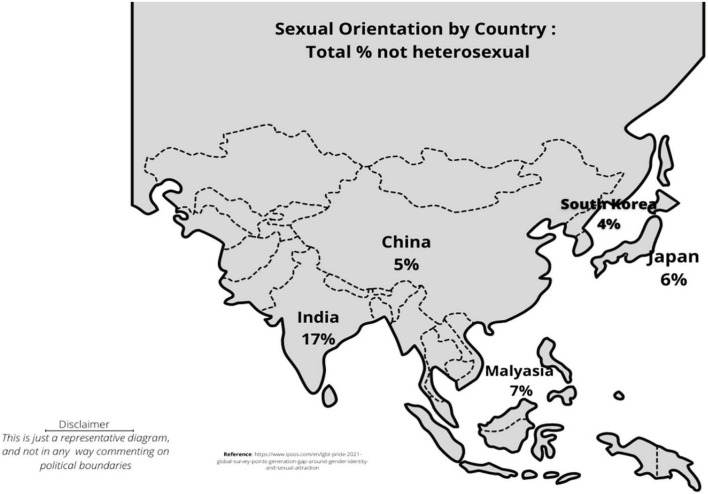
The percentage of individuals who identify as non-heterosexual in the Indian subcontinent according to LGBT + Pride 2021 Global Survey Report.

Figure 1 shows the percentage of individuals who identify as non-heterosexual in the different countries of the Indian subcontinent. However, there are limited data to adequately identify individuals who self-report their identity as not being heterosexual.

## Mental health of the LGBTQ youth

A practical paradigm for comprehending mental health disparities among LGBTQ people is Meyer’s Minority Stress model (MS model). MS model implies the stressors and their impact on sexual minorities. The stressors comprise objective external occurrences, expectations of these occurrences, and the internalization of negative societal attitudes. With the division of stresses into proximal and distal categories, the theory puts forth various aspects of the strains on LGBTQ individuals. It affirms the direct connection between the problems of LGBTQ individuals and environmental stress. It also highlights several aspects, i.e., prejudice events, stigma, disclosure, suicide, and mental disorders on the health of LGBTQ. Consequently, we attempt to illustrate several difficulties the LGBTQ youth in the Indian subcontinent experience that have a substantial influence on them (Meyer, 2003; Frost and Meyer, 2009).

### Role of adverse childhood events

Adverse Childhood Events (ACEs) refer to maltreatment, abuse and residing in an environment harmful to the development of a child. They tend to have traumatic and lasting negative impact on the health and well-being of the child (Sutter and Perrin, 2016; Boullier and Blair, 2018). Individuals exposed to greater than four ACEs have stronger association with sexual risk-taking, poor mental health, alcohol and drug abuse and self-inflicted violence (Hughes et al., 2017)It may also lead to the development of feelings of shame which itself has been associated with adverse health outcomes (Scheer et al., 2020).

In a survey conducted with LGBTQ youth between 14 and 18 years of age in the United States and Canada, 43% of the respondents reported being exposed to more than 4 ACEs, with higher scores in 9 out of 10 categories when compared to national samples (Craig et al., 2020). Additional studies from Canada, Mexico, Miami, and Israel show that female sex workers who identify as a sexual minority report having poor mental health compared to sex workers who do not identify as minorities, have a history of childhood trauma and suffer from mental health burdens (Cwikel et al., 2004; Ulibarri et al., 2009, 2013; Surratt et al., 2012; Puri et al., 2017). There is a growing body of literature indicating that LGTBQ individuals endure higher levels of traumatic experiences with a disproportionate impact on their physical and mental health.

### Role of bullying

LGBTQ youth are more likely to be victims of bullying (Juvonen and Graham, 2014; Earnshaw et al., 2017). LGBTQ victims of bullying in school report higher levels of absenteeism and lower academic achievement (Nishina et al., 2005). It has been linked to emotional distress, somatic complaints such as anxiety and headache, feelings of loneliness and self-blame as well as depression and anxiety (Gini and Pozzoli, 2013; Bruce et al., 2014).

### Role of unstable housing or homelessness

Households which follow heteronormative ideologies are a source of distress for the LGBTQ youth (Castellanos, 2016). Homeless LGBTQ youth report heteronormative attitudes, stigma, and discrimination at home which lead to significant physical and mental health concerns, including engagement in risky sexual behavior, substance abuse and family conflict due to disclosure of sexual orientation (Corliss et al., 2011; Rhoades et al., 2018; Baams et al., 2019).

Many LGBTQ youth are left homeless after disclosing their identity to parents and facing rejection (McCann and Brown, 2019). Studies show that 37% of people reaching out to crisis service providers experience homelessness and a large percentage of those in foster care or unstable housing are LGBTQ youth (Gangamma et al., 2008; Tyler, 2013). These LGBTQ youth face mental health issues like depression, anxiety, suicidal tendencies, substance abuse and show disparate school functioning (Gangamma et al., 2008; Tyler, 2013). Homeless LGBTQ youth are more likely to contract sexually transmitted diseases compared to homeless heterosexual youth and are more likely to report more internalizing symptoms, depression and suicide attempts compared to heterosexual youth (Tyler, 2013).

The coronavirus pandemic forced many LGBTQ youth to stay at home or return back home. This was a possible stressor for those individuals whose sexual orientation was not known at home. The college campus or other out-of-home environments may have been the first opportunity for such individuals where they could continue to explore their sexual identities. They might have participated in educational opportunities such as sexuality and gender studies classes to better understand their identities. However, taking such classes at home may be a cause of discomfort for their families affecting their continued ability to take such classes (The Chronicle of Higher Education, 2020).

### Role of religion

Religion plays an important role in societal evolution. This role extends to the acceptance of LGBTQ individuals in society. However, religion is often reported as a source of emotional distress by some of the LGBTQ youth. Most LGBTQ youth cite religion as a negative factor in their lives as their faith communities often show antagonistic behaviors toward same-sex relationships (Ream and Savin-Williams, 2005; Higa et al., 2014). Many LGBTQ youths (73% men and 43% women) may opt for “so called conversion therapy in an attempt to align with expected norms through personal righteousness, individual efforts, church counseling, psychotherapy, support groups, psychiatric treatment, family therapy, peer support and electroconvulsive shock treatments (Dehlin et al., 2015). Although these efforts may have some positive effects such as reduced depression and suicidal ideation, there is a lack of evidence on their effectiveness and they may also have adverse effects (Serovich et al., 2008; Dehlin et al., 2015).

### Role of self-harm, suicidality, and substance abuse

A study conducted in the US reported that 38.6% of the total LGBTQ participants had considered completing suicide while 27.9% of the participants had attempted suicide (Green et al., 2020). The rate of suicidality was higher among those who had undergone sexual orientation or gender identity conversion efforts (Green et al., 2020). Female LGBTQ youth report higher rates of self-harm and suicidality, as they are prone to face more abuse and violence; however, there is no difference in substance abuse among males and females (Russell and Joyner, 2001). Another study showed that LGBTQ adolescents in the grades 7–12 were twice as likely to complete suicide suffer from depression, alcohol, and substance abuse compared to their heterosexual peers (Russell and Joyner, 2001).

## Mental health problems faced by the Indian LGBTQ youth

There is a dearth of literature regarding the psychological issues faced by LGBTQ youth in India. However, extrapolating the difficulties faced by the adult population some basic assumptions can be made about the healthcare accessibility for adolescents (Rao and Jacob, 2012). A discord between gender identity and natal role, poor self and social acceptance of one’s sexual orientation, discrimination, physical, verbal and sexual abuse from family, friends, and peers during after coming out, fear of law-enforcement, loneliness, and lack of coping mechanisms lead to poor physical and mental health outcomes among the LGBTQ youth (Bhattacharya and Ghosh, 2020).

In Kolkata, a large proportion of the Hijra, Kothis and Transgender individuals (HKT) engage in traditional means of income (logon, cholla and badhai) due to the lack of proper education and employment opportunities, which can take a toll on their mental, physical, and sexual health. The HKT individuals that rely on cholla (begging on the streets and in trains) and logon (singing and dancing at weddings) are more prone to sexual abuse and survival sex (formally known as prostitution or sex work). A study among transgender individuals found that 16.7% of the participants had experienced sexual violence in the last 3 months. HKT individuals often report inaccurate health status, perhaps as a coping mechanism, although they claimed to be healthy but the PCS (Physical Composite Score) and MCS (Mental Composite Score) reflected otherwise (Bhattacharya and Ghosh, 2020).

Men who have sex with men (MSMs) suffer from depression due to the discrimination based on their sexual orientation, gender, physical or sexual violence, alcohol use, STIs and HIV status (Patel et al., 2015). One study reported a prevalence of 52.9% for psychiatric illness among MSMs according to the General Health Questionnaire (GHQ) which measures somatic symptoms, anxiety and insomnia, social dysfunction, and severe depression (Prajapati et al., 2014). Studies on a sexual minority of the Indian subcontinent have explored the explicit detrimental impacts of substance abuse on their mental health. Sexual minorities fall into a higher-risk population susceptible to substance abuse. With the involvement of a lot of physical illnesses, it overtly gives rise to a variety of mental health concerns (Tomori et al., 2016; Wilkerson et al., 2018; Wandrekar and Nigudkar, 2020). A study on women of sexual minority showed a high prevalence of alcohol abuse, Generalized Anxiety Disorder (GAD), depression, and suicidal risk among transgender individuals (Yr et al., 2018).

Among the adult gay population of Manipur, 6.2% of the participants suffered from GAD, 3.1% had panic disorders and 9.3% had social phobia (Niranjan, 2018). Similarly, mental health disorders were also found among individuals who identified as lesbians (Niranjan, 2018). In Bengaluru, among individuals 18 years and older who are the “Nirvana,” “Akwa,” “Kothis,” the mean depression and anxiety score was 8.5 and ranged from 6.1 among participants identifying themselves as “others” to 9.4 among the Nivana (Thompson et al., 2019). A significant statistical difference was also seen in reports of depression and anxiety based on gender identity. Nearly half of the Nirvana and Akwa hijra participants and a quarter of the Kothi participants reported physical violence in the previous 6 months and experiences of rape in the previous year mostly associated with younger age and working in Basti, sex work as Chelas or at a CBO (community-based organization) (Thompson et al., 2019). A majority of the studies in India focus on transgender individuals who are employed in survival sex including adolescents who are excluded by family and/or society and represent a marginalized community (UNDP, 2010).

## Mental healthcare for the Indian LGBTQ youth

Transgender adults and MSMs report avoiding free government healthcare and prefer to self-medicate or access private healthcare as they face discrimination due to social stigma (Patel et al., 2012; King, 2015). Sexual minority women avoid accessing mental healthcare due to the fear of mental health malpractice, the stigma of mental illnesses, and previous negative experiences (Ganju and Saggurti, 2017). Physician homophobia can be a deterrent for LGBTQ patients to seek healthcare (Bowling et al., 2016). Even though healthcare providers are developing positive attitudes, discriminatory practices like delayed services and judgmental attitudes using direct and indirect verbal indicators inhibits the LGBTQ community from accessing healthcare services (Agoramoorthy and Minna, 2007).

Senior psychiatrists have been found to discriminate against LGBTQ individuals based on the traditional gender stereotypes, leading to reduced access to high quality healthcare services (Chakrapani et al., 2011). Despite the efforts of the WHO and the Indian Psychiatric Society to non-pathologize homosexuality, many healthcare professionals still carry out unethical practices such as “so called conversion therapy based on social stigma (Rao et al., 2016). Blood banks are being scrutinized as some of them discourage or “ban” the donation of blood by LGBTQ individuals according to the Central government’s guideline for Blood Donor Selection and Blood Donor Referral that was brought into effect in the 1980s to control the global AIDS pandemic (Kalra, 2012; Hindustan Times, 2021).

Some Indian states, Tamil Nadu being the first, have framed transgender welfare policies under which transgender individuals can access free sex reassignment surgery with proper documentation (UNDP, 2013). A survey among medical students showed that students with greater knowledge about homosexuality showed positive attitudes toward LGBTQ individuals (Banwari et al., 2015). Women had a more positive attitude, which did not correspond with greater knowledge in the study (Madhan et al., 2012). Dental students were less informed and thus, lacked positive attitudes toward LGBTQ patients. This indicates the need for an all-inclusive non-discriminatory curriculum focused on problems of the LGBTQ individuals. Academic books used by the medical students are also seen to contain negative stereotypes regarding LGBTQ individuals which promote social stigma among young medical students (Chatterjee and Ghosh, 2013). A psychiatry book used by the West Bengal University of Health Sciences contains terms such as cross-gender homosexuality and ego-dystonic homosexuality. A forensic medicine book states that homosexuality is an offense, and such individuals may pose a social, moral, and psychological problem. However, the National Medical Commission recently released an advisory to amend the discriminatory information in medical textbooks, taking a step toward LGBTQ inclusion in the medical fraternity (Meyer, 2003).

The health disparities faced by the LGBTQ community in India due to discrimination and exclusion reflects the deeply embedded cultural practice of homophobia and transphobia, supported by a lack of adequate legal protection against discrimination based on sexual orientation and gender identity.

## Recommendations for improvement of healthcare services for LGBTQ youth in India

First, strategies must be developed from the ground up to tackle this issue. Childhood experiences profoundly shape our future. Considering the predominance of unfavorable childhood events among LGBTQ youth, a secure and supportive upbringing might lessen their mental health burden to a great extent. An effective strategy for addressing childhood issues should be started with proper conditioning of schools since this is the primary setting where most children learn about social mores and standards (Willging et al., 2016a). The LGBTQ youth and the SHPs agree that there is a need to increase knowledge, improve skills, and change attitudes toward LGBTQ-related topics among SHPs (Reisner et al., 2020). Elimination of the bullying culture could be an impactful approach owing to it having a severe negative impact on victims. In the Indian subcontinent, there is a dearth of effective prevention strategies to deal with bullying toward LGBTQ youth; this problem has instilled panic in LGBTQ youth for ages. To overcome this challenge, training School Health Professionals (SHPs) can be helpful as they would be enabled to serve as a source of care and support for LGBTQ youth. SHPs need to address bullying and victimization to help prevent poor psychological outcomes among LGBTQ youth.

Unstable housing is one of the major challenges LGBTQ youths encounter in the Indian subcontinent. With the provocation of many problems, mental health has also drastically deteriorated due to homelessness or domestic disputes. While family support could be a big comfort for LGBTQ youth, their rejection instead makes it challenging for LGBTQ youth to live with family. If the family members were enlightened and empathetic toward LGBTQ youth, the quality of their life could substantially improve. Therefore, our recommendation in this regard is to educate and involve family members to improve the lives of LGBTQ youth. We must identify ways to safely involve family members who are vital to the LGTBQ individuals’ lives. If a welcoming environment could be established in the family for this vulnerable population, it could relieve many of the mental health issues (Meyer, 2003; Pufahl et al., 2021).

Homophobic hospital settings have been one of the omniscient worries for most of the LGBTQ youth. Though they have been battling several mental health issues for years, they are hesitant to get help from healthcare providers due to cultural baggage. They have many unaddressed health concerns they fear disclosing, which keeps inflating their health. If the clinical setting environment could be improved, it would aid in resolving both biological and mental health difficulties. In enhancing healthcare services for LGBTQ youth in India, it is essential to better understand the unmet medical needs of LGBTQ individuals and develop competencies among healthcare workers to handle such patients with compassion and sensitivity.

In light of this pressing issue, our recommendations skew toward training healthcare professionals to provide appropriate care to LGBTQ youth. Taking into consideration the six building blocks of the CanMEDs 2015 Physician Competency Framework of the Royal College of Physicians and Surgeons of Canada, the health inequities of the LGBTQ population in India can be addressed by psychiatrists with a range of skills to work with varied patients to achieve a complete understanding of youth predicaments, advocacy, and leadership within communities (Frank et al., 2015; Kealy-Bateman, 2018). LGBTQ–informed treatment involves easily implementable additions to practices like asking the clients about the pronouns they prefer, avoiding disrespectful language, and not making assumptions about the health risks and stereotypes associated with LGBTQ individuals (Frank et al., 2015; Kealy-Bateman, 2018). It has been seen that simply inquiring about the sexual identity of a person decreases the probability of treatment dropout by 30% (Mandal and Dhawan, 2018).

Consequently, healthcare providers must be trained to create a welcoming environment for this vulnerable population. A study by Saahas (meaning courage), a non-profit organization in India, showed that LGBTQ participants showed positive attitudes toward LGBTQ-affirmative treatments. The primary reason for reaching out to Saahas was to discuss queer-specific experiences related to stigma and relationship challenges, such as dealing with family members. Using preferred pronouns and identity validation made the LGBTQ participants more willing to seek help at the organization. In particular, they discussed the multiple types of issues that affect LGBTQ youth, including impacts of religion, caste, class identities, differentiation between environmental problems and those arising from dysfunctional thoughts, and the absence of being in an open space to reveal and discuss the manifestations of sexual abuse and victimization (Wandrekar and Nigudkar, 2019).

Furthermore, healthcare services for LGBTQ youth must be based on the best evidence for clinical effectiveness. Ratner’s 12-step recovery model for homosexual alcohol abusers proved effective and is used by LGBTQ individuals (Ratner, 1988). The reason Ratner’s program worked is that it makes a conscious effort to differentiate between a lesbian and gay lifestyle and the effects of addiction in both (Wandrekar and Nigudkar, 2019). The model also considered possible addiction-related issues like sexual abuse, grief, and victimization (Ratner, 1988). It has been seen that four sessions of motivational interviewing (MI), as compared to Cognitive Behavioral Therapy (CBT) and MI for alcohol-dependent MSM, can reduce drinking for a sustained time period (Morgenstern et al., 2007). Community Reinforcement Approach (CRA) has been seen to be effective for homeless LGBTQ youth (Grafsky et al., 2011).

Transparency, effective communication, and extension of community health resources to these vulnerable communities are fundamental to ensuring their overall health and wellbeing.

Although LGBTQ youths have been associated with many undesirable health concerns like more serious self-harm attempts, depression, and substance abuse disorders, there is no standard and reliable source to understand their health issues. Standardized statistics on LGBTQ health status would be eye-opening here since it could help draw attention to the areas that require it the most. In India, there is a scarcity of research related to the understanding of the mental health needs of LGBTQ individuals, and therefore, there is a lack of protocols in place to address gaps in their care. Although some practitioners have established LGBTQ-friendly practices, systematic documentation of these practices is needed with the participation of members of different health professions to develop guidelines providing holistic and gender-affirmative healthcare services (Ranade and Chakravarty, 2013).

In this context, we recommend focusing on developing standardized methods for collecting information about the health status of LGBTQ youth. In doing so, the APA (American Psychological Association) LGB (lesbian, gay, and bisexual) guidelines provide an example of evidence-based guidelines on the management of psychological issues and their determinants for LGB individuals (American Psychological Association, 2021).

Although LGBTQ people’s past experiences led them to view religion as a menace to their lives, it has the potential to save LGBTQ youth if implemented positively. Religious communities can play an important role in the management of mental health issues being faced by LGBTQ youth. Some religious communities provide support to LGBTQ individuals such as the provision of anti-suicide counseling and resources for homelessness with most LGBTQ individuals displaying a preference for in-person resources (Raedel et al., 2020). If implemented In the Indian subcontinent, its adaptation might aid in solving many mental health concerns of LGBTQ youth.

Social media, which possesses a great capacity for transformation, can spell its magic cast on the LGBTQ community if utilized in an effective direction. Previous literature has indicated the use of social networking sites (SNSs) among LGBTQ youth has led to the creation of a community with some level of social support and increased access to sexual health-related information which contributes to positive identity development (Gray, 2009; DeHaan et al., 2013; Craig and McInroy, 2014; Fox and Ralston, 2016). While they are coping with various psychological issues, social community support can help ease the load of addressing it rather alone. Therefore, the use of social media to create a sense of community among LGBTQ youth should be encouraged. However, some of these SNSs have also facilitated cyberbullying or discrimination and victimization based on their sexual orientation and gender identity, which has led to psychological distress and worse mental health outcomes among LGBTQ youth (Varjas et al., 2013; Fox and Moreland, 2015; McConnell et al., 2017; Abreu and Kenny, 2018).

Those who are experiencing a mental breakdown may find great comfort in connecting with a peer who has gone through comparable experiences. A positive shift in the delivery of LGBTQ mental healthcare might result from the formal incorporation of peer intervention in the healthcare setting. With the exploration of many underreported physical and mental health concerns, it can provide remedies to LGBTQ individuals at a root level. It can also create a sense of empowerment with recognition of their necessities (Willging et al., 2016b; Worrell et al., 2022). Therefore, to achieve a positive transformation in LGBTQ mental healthcare, our recommendation leans toward the incorporation of a peer intervention in healthcare delivery.

Our last contribution to the list of recommendations is the digitalization of healthcare settings. This endeavor of comprehensive service delivery may be made achieved through the inclusion of technology. The recent “LGBT Health and Wellness Cloud” initiative in Bengaluru has demonstrated the potential of technology for global healthcare services (The New Indian Express, 2022). If such models are implemented throughout the nation, it can open the door of healthcare services to many underserved LGBTQ people (Nisly et al., 2018). We thus advocate for the alignment of technology in healthcare with a view to raising the bar for equity.

## Conclusion

This article highlights the various aspects in which the LGBTQ community has been marginalized within the complex Indian medical system. There is a vital need for reliable centralized statistical data regarding the number of individuals identifying as LGBTQ in India, their healthcare needs as well as the barriers and facilitators of care. As gaps in healthcare demands and accessibility are identified, remedial measures can be taken to ensure the availability of high-quality healthcare services for this traditionally marginalized group. Due to the diversity of this community, reliable collection of accurate information may seem challenging. However, without adequate resource allocation, the medical and mental health needs of a large proportion of this population are being missed or under-identified.

Healthcare professionals must be trained to adequately interview and provide their services to LGBTQ individuals, with compassion and acceptance for all human experiences. Through high quality, evidence-based medical training, health professionals will be better equipped to provide a holistic care. Professional and political organizations should develop policies to allow for the provision of easily accessible healthcare services in an inclusive medical environment for the LGBTQ community. Ultimately, these health disparities must be eliminated as they have the potential to impact the overall health of the community as no group exists in isolation and the health of a community is defined by the health of all those that inhabit it.

## Author contributions

LG, PG, SS, and VA: conceptualization. LG, PG, SS, SA, and NS: investigation. LG, PG, SA, and PO: methodology. LG, PG, and SS: resources. LG: software. LG, PO, SA, VA, AUH, and DD: supervision. LG, PG, SS, AG, AH, and NS: validation. LG, PG, SS, AG, PO, and SA: visualization. PG, SS, LG, and NS: writing – original draft. All authors: data curation, writing – review and editing.
